# The Case for Vinegar Ingestion

**DOI:** 10.3390/nu18152574

**Published:** 2026-08-06

**Authors:** Carol S. Johnston

**Affiliations:** Nutrition Program, College of Health Solutions, Arizona State University, Phoenix, AZ 85004, USA; carol.johnston@asu.edu

**Keywords:** vinegar, acetic acid, acetate, acetyl-CoA, AMPK, deacetylation

## Abstract

Acetate is emerging as an influencer of wellbeing. The interest in acetate parallels the mounting reports of preclinical and small-scale clinical investigations over the past two decades suggesting the health benefits of vinegar, a dietary source of acetic acid along with fermented and pickled foods. Additionally, acetic acid is generated during fermentation by the gut microbiome and is considered a main benefit of the gut microbiome. However, numerous factors adversely impact acetic acid production by gut microbiota such as medications, disease states, and low fiber intakes. Since acetic acid rapidly deprotonates when entering the blood stream, it is a source of systemic acetate. A third source of acetate is endogenous production by various tissues via deacetylation reactions, and endogenous acetate production is 2-fold that generated by microbiota fermentation. This narrative review highlights acetate metabolism and impacts on health parameters as well as the role of vinegar in health promotion as an exogenous source of acetic acid.

## 1. Introduction

**Figure 1:**
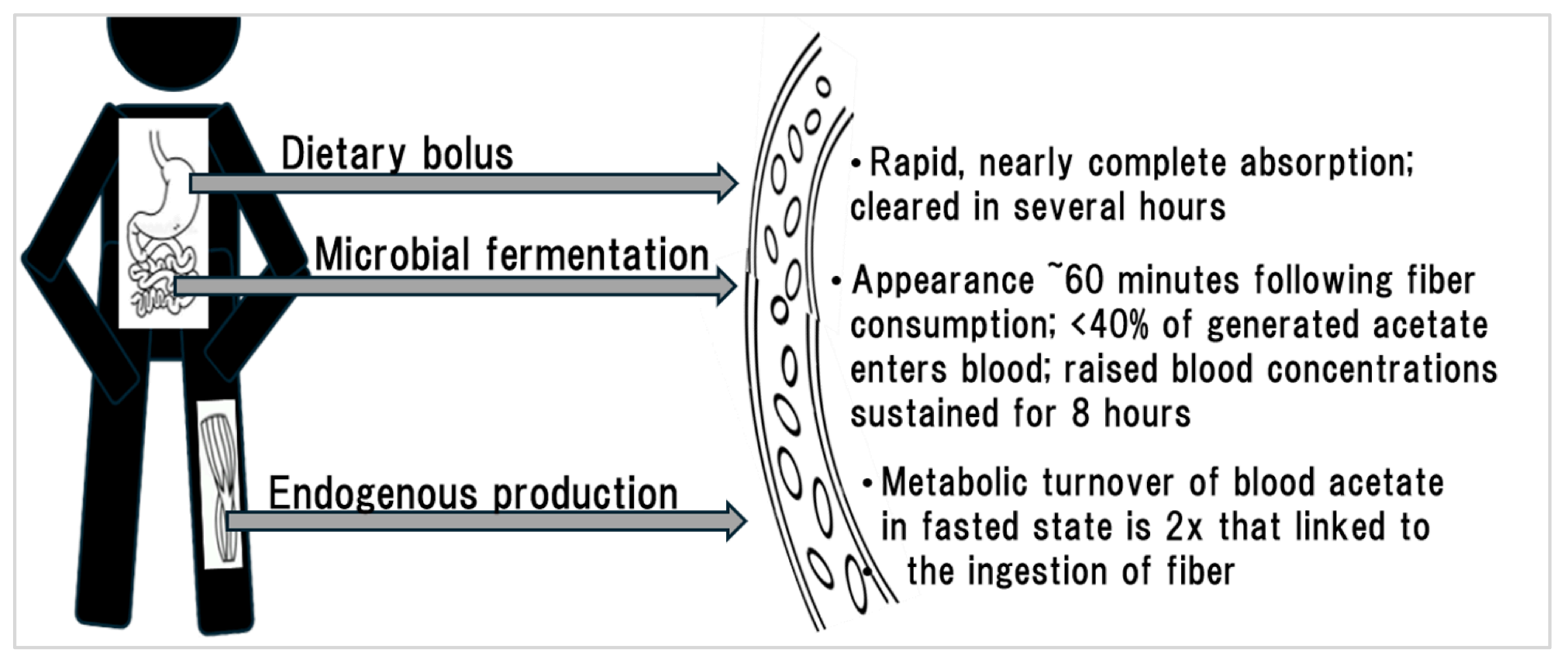
Conceptual summary of the three sources of blood acetate. Dietary dose in published reports range from 0.5 to 3.0 g acetate/acetic acid. Estimated generation of acetic acid from microbial fermentation following 20 g fiber ingestion: 7.5 g. (Data sourced from [4,6]).

Acetate, the conjugate base of acetic acid, is emerging as a significant influencer of wellbeing. The interest in acetate parallels the mounting reports of preclinical and clinical investigations over the past two decades demonstrating a myriad of health benefits for vinegar ingestion [1,2,3]. Vinegar is the fermented product of juice sourced from carbohydrate-rich foods (e.g., fruit, vegetables, or grains); the carbohydrates are converted to ethanol by yeast and further fermented by bacteria to yield acetic acid. Vinegars are an excellent source of acetic acid, and commercial vinegars are typically 5% acid and contain 750 mg acetic acid per tablespoon. Dietary acetic acid is rapidly absorbed beginning in the stomach, and upon entering blood (pH 7.4), acetic acid loses a proton and transforms into acetate (Figure 1).

In healthy adults, following the ingestion of a single dose of sodium acetate (providing 1.8 g acetate), acetate concentrations in plasma nearly doubled at 15 min, peaked at 45 min, and returned to fasting levels within two hours [4]. Acetic acid is also generated during fermentation by the gut microbiome, along with other short-chain fatty acids (SCFA), primarily propionic acid and butyric acid (Figure 1). The amount of microbiota-derived acetic acid entering plasma from 20 g dietary fiber has been estimated to be approximately 3 g in humans [5,6], which is less than 40% of the amount generated in the gut [7,8].

Contrasting with the absorption kinetics of an oral dose of acetate, acetate sourced from fermentation of ingested fiber in healthy adults appeared in plasma slowly, peaking at 150 min with elevated levels maintained for about eight hours [4]. However, numerous factors may impact the production of SCFA by gut microbiota including medications (e.g., antibiotics, metformin, statins, plus others), genetics, disease states, and the fiber intakes [9,10]. The fluctuations in production rates can vary considerably and are context-based.

Microbial production of the three primary SCFA is a chief feature of the gut microbiome. Based on human fecal analyses, the acetate-to-propionate-to-butyrate ratio approximates 60:20:20 [11]. (Note that fecal SCFAs are often measured as a proxy for cecum levels, but fecal levels represent the residual fractions of the SCFAs following extensive metabolism and absorption across the length of the colon.). In portal blood this ratio shifts to 82:9:9 and in arterial blood, to 94:2:4 [11]. These data suggest that in humans there is heightened colonic metabolism of propionate and butyrate in comparison to acetate as well as a more effective hepatic uptake. Thus, a greater proportion of microbial-derived acetate is released into portal blood and eventually into the general circulation in comparison to propionate and butyrate.

A third source of acetate is the endogenous production by various tissues via deacetylation reactions (Figure 1). Using stable isotope approaches in healthy adults, reports suggested that endogenous acetate production is approximately 6–7 µmol·kg^−1^·min^−1^ in fasted, rested adults [6,8]. Conversely, endogenous acetate production following a 20 g bolus of lactulose, averaged over 6 h, was about one-half that for endogenous acetate production, 3.2 µmol·kg^−1^·min^−1^ [6,8].

Acetate metabolism and impacts on health parameters, while considering the effects of vinegar ingestion on these same parameters as an exogenous source of acetic acid, is a broad, unexplored topic and the focus of this narrative review. The biomedical literature was searched (PubMed) for articles in English published up to June 2026 using the search terms ‘acetate’, ‘acetic acid’, and ‘vinegar’ in titles and abstracts with particular attention to preclinical and clinical studies encompassing metabolic and health outcomes. Additionally, the reference lists of eligible studies were screened, and relevant review articles, systematic reviews, and meta-analyses were included. Online searches were done using an internet search engine (Google) with the same terms to identify other sources of applicable scientific information. All the relevant abstracts were screened by the author, and studies that did not describe metabolic or health outcomes influenced by acetate, acetic acid, or vinegar were excluded from the pool of results. Preference was given to studies providing high-quality experimental evidence and to clinical investigations using randomized controlled trial (RCT) study designs, which can provide causal proof. Yet, such studies are conducted in highly controlled environments and results are not necessarily evidence of efficacy in real-world practice. Synthesis of the information from the studies reviewed suggested four predominant metabolic processes (with overlap)—energy production, fat oxidation, glucose disposal, and brain health—impacted by endogenous acetate, microbial derived acetic acid, and ingested vinegar, which are reviewed below.

## 2. Energy Production

Mitochondria, termed the ‘powerhouse’ of cells, are cellular organelles that generate high-energy ATP (adenosine triphosphate) to power cellular activity including muscle contraction, chemical synthesis, nerve impulses, active transport of molecules across membranes, and cell division. In mitochondria, ATP is generated through oxidative phosphorylation subsequent to the TCA cycle (tricarboxylic acid cycle), a process requiring coenzyme A (CoA). Acetyl-CoA is formed predominantly from pyruvate (derived from glucose), but also from acetate coupled to CoA, a reaction catalyzed by site-specific acyl-CoA short-chain synthetases. This latter pathway is increased in muscle, heart, and brain astrocytes [12]. In kinetic experiments on healthy adults (*n* = 6; 20–28 years of age; 20.5 kg/m^2^) using constant infusions of [1^−13^C]acetate, the oxidation rate of acetate accounted for 6.5% of overall energy expenditure at rest in the fasted state [13].

**Figure 2:**
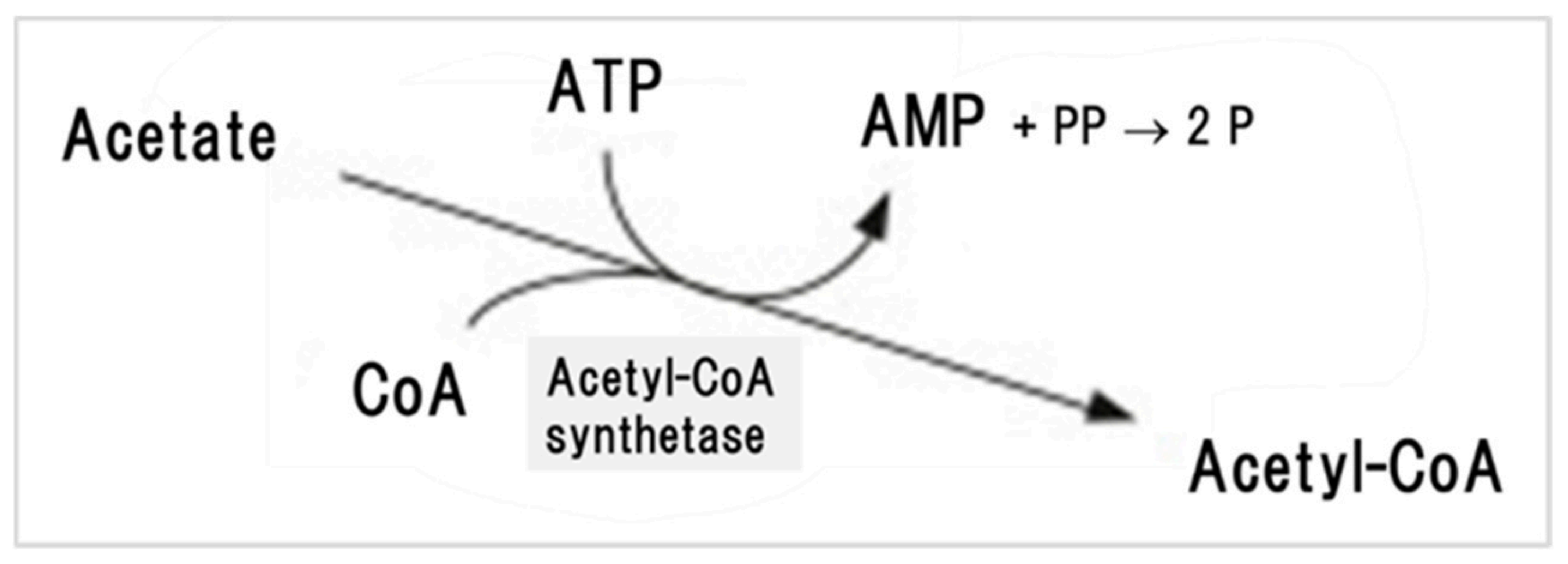
The conversion of acetate to acetyl-CoA catalyzed by the enzyme acetyl-CoA synthetase.

Data from several small RCTs in adults suggest that acetate signals for enhanced fat oxidation via the TCA cycle as well as the storage of glucose as glycogen in liver and muscle. Van der Beek et al. reported that distal colonic acetate infusions in healthy overweight men (*n* = 6) increased fat oxidation by 25% compared to no change in the placebo group after two hours [14]. In a follow-up study, these same investigators infused SCFA mixtures into the rectum of healthy, normoglycemic overweight men (*n* = 12) in the fasted state and observed significant increases in energy expenditure and fat oxidation over the next two hours, changes that were directly correlated to the change in plasma acetate but not to changes in plasma propionate or butyrate [15]. It is not the oxidation of acetate per se driving these changes, rather the data suggest that acetate is altering the AMP (adenosine monophosphate)/ATP ratio when converted to acetyl-CoA (Figure 2), and that this possible rise in the AMP/ATP ratio is activating a key regulator of metabolism, AMPK (AMP-activated protein kinase) [16].

**Figure 3:**
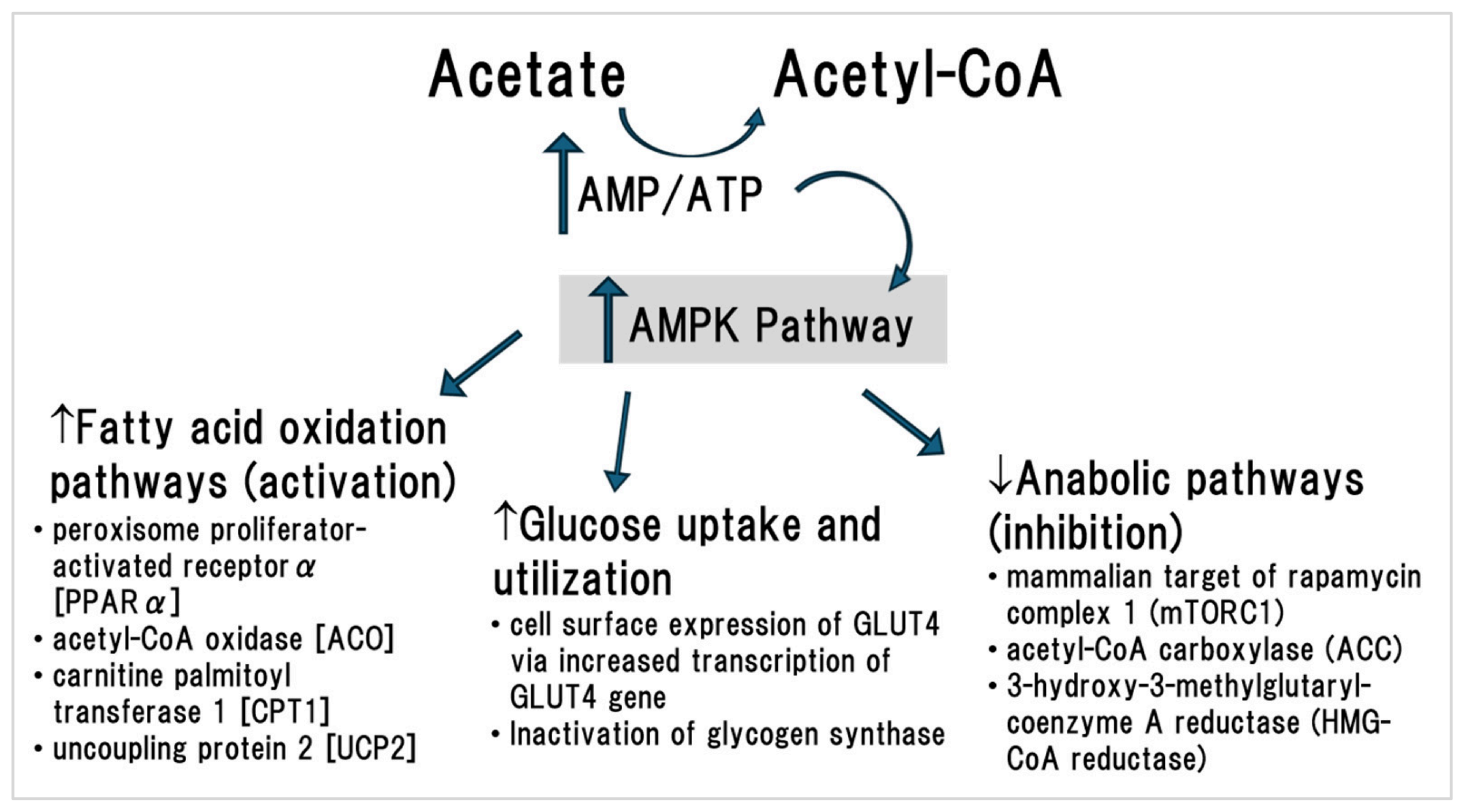
Proposed metabolic consequences of AMPK activation and the enzymes involved. (Data sourced from [18,21,22]).

AMPK ‘senses’ energy status in cells by directly binding adenine nucleotides and functions to restore energy balance. Elevated AMP/ATP ratios signal a low energy status, and the binding of AMP to an AMPK subunit facilitates the phosphorylation/activation of AMPK, which in turn redirects metabolism to stimulate ATP production and reduce ATP utilization (see detailed review by Herzig and Shaw [17]). AMPK activates lipases to release fatty acids from adipose stores to feed into the TCA cycle for ATP synthesis (Figure 3). Glucose uptake from blood is enhanced via AMPK-induced translocation of glucose transporter type 1 and 4 (GLUT1 and GLUT4) to cell membranes promoting glucose disposal (discussed below). AMPK also inhibits lipid synthesis, gluconeogenesis, and protein synthesis to limit ATP consumption [18]. In preclinical trials, orally administered acetate (10.5 mg/kg) raised the AMP/ATP ratio in rat abdominal muscle nearly 3-fold after 2 min, and an increase in phosphorylated AMPK was noted in muscle at 3- and 10 min following acetate injection [19]. In aging rats, oral acetic acid injection (50 mg daily for 20 weeks) raised levels of phosphorylated AMPK in the soleus muscle in comparison to the control rats receiving only water injections [20]. Acetic acid treatment also attenuated age-related reductions in oxygen consumption and energy metabolism and decreased intramuscular lipid accumulation in comparison to control rats [20]. Although these pre-clinical trials are promising, adequately powered, long duration RCTs in patient populations and healthy adults are necessary to fully understand the actions of AMPK in health promotion.

## 3. Fat Oxidation

In rat and bovine hepatocytes, acetic acid treatment increased AMPK phosphorylation and upregulated the expression of lipid oxidation genes and reduced the expression of lipogenic genes [23,24]. In vivo, Kondo et al. observed significant reductions in body weight gain (−26%) and fat mass gain (−23%) in high-fat-fed mice administered 0.3% or 1.5% acetic acid intragastrically for 6 weeks compared to the sham controls [22]. Hepatic gene expression was significantly upregulated by 16–60% for several enzymes involved in fatty acid oxidation in the acetic acid-treated mice versus controls (e.g., peroxisome proliferator-activated receptor α [PPARα], acyl-CoA oxidase [ACO], carnitine palmitoyl transferase 1 [CPT1], and the thermogenic protein, uncoupling protein 2 [UCP2]) [22]. To address the role of AMPK activation in these findings, the investigators demonstrated that the knockdown of α2 AMPK gene expression in a human hepatoblastoma cell line eradicated the effect of acetic acid treatment on PPARα, ACO, CPT1, and UCP2 gene expression [22].

Under controlled conditions, Liu et al. examined the impact of subcutaneous acetate injection (2 g/kg for four days) on lipid metabolism in liver, leg muscle and adipose tissue in rabbits [25]. In skeletal muscle, acetate treatment significantly increased the mRNA levels of CPT1 and PPARα, as well that of fatty acid-binding protein and fatty acid transport protein, compared to control groups. Additionally, gene expression of CPT1 and PPARα were significantly raised in adipose tissue of the rabbits injected with acetate compared to controls; however, in the liver, PPARα gene expression decreased while CPT1 gene expression increased with acetate treatment [25]. Phosphorylated AMPK was significantly elevated in liver and adipose tissue, but not leg muscle, with acetate treatment, and acetate treatment reduced both hepatic and intramuscular lipid content relative to controls [25].

In an RCT that enrolled 155 healthy overweight adults (25–60 years of age), Kondo et al. examined the impact of vinegar intake (0, 750, and 1500 mg acetic acid daily for 12 weeks) on body fat indices [26]. Cross-sectional abdominal visceral fat area (VFA) and subcutaneous fat area (SFA) were measured by computed tomography at the level of the fourth to fifth lumbar vertebrae. After 12 weeks, significant reductions in both VFA and SFA were noted for both acetic acid groups versus the control group, and the total fat area was reduced by 3.5% in the participants receiving the highest dose of acetic acid compared to a 1.0% rise total fat area for the control participants [26]. Total body weight decreased 1.2–1.9 kg in the acetic acid groups compared to a 0.4 kg increase in the control group. Based on their previous preclinical data in high-fat-fed mice, the investigators postulated that acetic acid ingestion stimulated hepatic fatty acid oxidation via AMPK activation leading to reductions in visceral and subcutaneous fat volumes, as well as total body weight [26]. However, this link remains conceptual since AMPK regulation of adipocyte lipolysis is complex and can include inhibitory effects on hormone-sensitive lipase depending on biological context.

Additional RCTs directly examining vinegar/acetic acid ingestion, adiposity, and body weight are scarce. Alternatively, studies on high fiber diets could be viewed as associative evidence for a link between acetic acid ingestion and adiposity since these diets stimulate microbial fermentation (which elevate endogenous acetate levels). It is well documented that adoption of high fiber diets is related to lower body weight and weight loss. In a 2020 systematic review and meta-analysis (62 human trials, *n* = 3877), Jovanovski et al. concluded that the addition of viscous fiber to an ad libitum diet (median dose of 8 g viscous fiber/d and median duration of 8 weeks) reduced body weight (−0.33 kg; 95% CI: −0.51, −0.14 kg; *p* < 0.05) compared with the control treatment [27].

With a focus on the gut microbiome and SCFA levels, Mayengbam et al. conducted an RCT in overweight/obese adults (*n* = 53) to examine the physiological effects of a high-fiber intake (15 g pea fiber daily for 12 weeks) [28]. Daily pea fiber ingestion was associated with a modest change in the gut microbial profile as evidenced by a significant increase in the abundance of Lachnospira. A rise in fecal acetate in the high-fiber group was the only significant change in SCFA during the study, and the rise in acetate was directly correlated with Lachnospira abundance. Moreover, body weight change during the study was inversely related to Lachnospira abundance [28]. Bang et al. observed similar findings in vitro using human fecal microbiota as the fermentation model [29]. The most prominent change in fecal microbial composition during pectin fermentation in this model was an increase in Lachnospira abundance. Moreover, acetate concentrations rose significantly after 6–12 h of incubation while there was little change in propionate or butyrate in the system over the same period [29].

## 4. Glucose Disposal

AMPK activation also stimulates cellular glucose uptake and oxidation (Figure 3). The glucose transporter GLUT4, which is expressed primarily in skeletal muscle and adipose tissue, is responsible for the transportation of glucose across cell membranes [30]. When stimulated, intracellular GLUT4 storage vesicles undergo exocytosis to the plasma membrane and function in the rapid uptake of glucose into the cell. Insulin’s role in glucose disposal occurs mainly through insulin-stimulated GLUT4 translocation to the cell surface; however, acetate also stimulates this process via AMPK activation. A time-course study in rat L6 myoblasts demonstrated that 0.5 mM acetic acid treatment increased cellular AMP levels within 2 min, the phosphorylation of AMPK within 5 min, and the cell surface expression of GLUT4 within 10 min after the addition of acetic acid [21]. Pretreatment of the cells with AMPK inhibitors (compounds C or araA) fully suppressed these acetic-acid induced changes [21].

In hyperglycemic obese OLETF rats, acetic acid injections (52.5 mg/kg for 27 weeks) did not impact mRNA levels of lipolytic genes in abdominal muscle compared to the water-injected control OLETF rats; however, acetic acid treatment increased transcripts of myoglobin and GLUT4 genes in abdominal muscle of the OLETF rats [27]. Further analyses demonstrated a 3-fold increase in the AMP content in the abdominal muscle, which was related to enhanced AMPK phosphorylation within 3 min of the administration of acetic acid [31]. These results conflicted with those of Liu et al., discussed above, where acetic acid administration did not activate AMPK in rabbit leg muscle [25]. This discrepancy may relate to specific muscle characteristics. Park et al. demonstrated muscle-specific effects of acetic acid supplementation in estrogen-deficient ovariectomized female rats with daily access to a running wheel to promote voluntary exercise for 13 weeks [32]. (Note, estrogen activates AMPK via the calcium/ calmodulin-dependent protein kinase 2 [33].) No significant acetate-driven differences were noted in the ratio of phosphorylated AMPKα to AMPKα in soleus muscle at the end of the trial; whereas, this ratio was significantly elevated in gastrocnemius muscle. This difference in AMPK activation may be related to the differing fiber types between muscles [32].

In active adults with type 2 diabetes, who were otherwise healthy and not on medications, Mitrou et al. examined the effect of acetic acid ingestion on glucose disposal by catheterizing the radial artery and a contralateral antecubital vein of the forearm muscles [34]. In this crossover RCT, participants ingested 1.8 g of acetic acid as vinegar diluted in water and a test meal containing 75 g carbohydrate or placebo. Blood glucose changes were monitored for 5 h post meal. Glucose uptake by the forearm muscles during the postprandial period increased significantly (+32%) during the acetic acid treatment phase as compared to the control treatment phase [34]. Additionally, postprandial hypertriglyceridemia decreased significantly (−9%) during the acetic acid treatment phase versus control. Potential mechanisms for this enhanced glucose disposal during acetic acid treatment were not examined in this study, and several mechanisms may contribute to these findings such as delayed gastric emptying or insulin effects; but these data are compatible with the preclinical data implicating AMP activation by acetate leading to increased GLUT4 expression at the muscle cell surface.

The favorable impact of vinegar ingestion on blood glucose has been documented in both healthy adults and patients with type 2 diabetes over the recent decades. Four separate meta-analyses in adults uniformly concluded vinegar ingestion may be a useful adjunctive tool for improving glycemic control [35,36,37,38]. The most recent meta-analysis (25 studies encompassing 1320 participants) demonstrated significant reductions hemoglobin A1c (−0.91%; 95% CI: −1.62 to −0.21), a widely applied measure for assessing the average amount of glucose in the blood stream over the past three months. The meta-analyses demonstrated that the reductions in hemoglobin A1c were most pronounced in obese or diabetic patients [36]. However, the risk of bias was high for twelve of the studies, particularly in terms of randomization and blinding, and the significance of these results was impacted by the sensitivity analysis [36].

## 5. Brain Health

### 5.1. Mitochondrial Energetics

Given the role mitochondria play in powering cellular activities, and the fact that the brain is one of the most metabolically active organs, it is plausible that acetate may influence brain metabolism. Astrocytes, which comprise up to 40% of total brain tissue volume, utilize acetate for up to 20% of energy needs at rest based on magnetic resonance spectroscopy after intra-venous 1-^13^C labeled acetate infusion in fasted humans (*n* = 4) [39]. In healthy male Sprague–Dawley rats, a single oral dose of acetate (glyceryl triacetate; 6 g/kg body weight) significantly increased brain phosphocreatine (PCr) levels and the PCr/Cr ratio at 4 h compared to control; however, brain ATP levels were unchanged [40]. (Note that this oral dose of acetate is 20–200-fold greater than that used in clinical trials [26,34]; yet this dosage did not result in any detectable toxicity in the rat model [41].) PCr is a high-energy phosphate reservoir that can generate ATP at a higher rate than oxidative phosphorylation [42]. PCr maintains brain ATP levels by offering ‘stress-adaptation capacity’ at times of high energy needs (e.g., sleep deprivation, mental fatigue associated with learning/memory, psychiatric illnesses that involve brain bioenergetic deficits) [43,44].

Acetate may also promote oxidative phosphorylation directly. In ex vivo isolated microglia from germ-free mice, Erny et al. ran a series of experiments establishing that microglia metabolic functions are controlled by acetate [45]. Specifically, complex II, the key mitochondrial enzyme linking the TCA cycle and the electron transport chain, was impaired in germ free mice, an insult that was reversed by acetate [45]. Impaired brain energy metabolism, as indicated by reduced ATP levels and disrupted activity of key mitochondrial enzymes, is a prominent feature of traumatic brain injury [46,47]. Arun et al. reported that the impairment in brain energy metabolism due to a cortical impact injury was partially reversed by acetate supplementation in male Sprague–Dawley rats as evidenced by a 2.5-fold increase in ATP levels in the affected brain region at four days post-injury in comparison to controls [48]. The authors proposed that acetate conversion to acetyl-CoA compensates for reduced enzyme activity by directly providing a key metabolite promoting oxidative phosphorylation [48].

In 5xFAD mice, a transgenic model for Alzheimer’s disease (AD), Böswald et al. examined the effects of inulin supplementation on the microbiota–gut–brain-axis [49]. After seven weeks of daily supplement ingestion (1 cm gelatinous pellets containing 1.6 g fiber provided ad libitum), the typical rise in amyloid-beta precursor protein and beta-secretase 1, the rate limiting enzyme in amyloid-beta protein production, was nearly undetectable in brain tissue from the fiber-supplemented mice in comparison to AD control mice. Colonic SCFA levels were significantly increased in the fiber-supplemented AD mice relative to the AD control mice, a change attributed to elevations in acetate as this was the only colonic SCFA that differed between groups over the course of the study [49]. Although Böswald et al. attributed the beneficial effects to acetate, they did not elucidate mechanisms for the reduced plaque load in the inulin-fed mice. Mitochondrial dysfunction and reduced ATP production, conditions which acetate has been suggested to reverse, have been linked to plaque counts at autopsy in patients with confirmed AD and in animal models of AD [50,51].

Further, recent evidence suggests that acetate participates in metabolic coupling between astrocytes and neurons. In a mouse model of middle cerebral artery occlusion, Peng et al. demonstrated that, relative to saline-treated controls, oral acetate supplementation (2 mg sodium acetate/g body weight for 28 days) enhanced glucose uptake by astrocytes as well as the activation of the astrocyte–neuron lactate shuttle, a system that provides astrocyte-derived lactate to neurons to fuel the TCA cycle [52]. Lactate accumulation in neurons was also demonstrated to stimulate protein lysine lactylation, which regulates gene expression by modifying histones. In the acetate supplemented animals, brain injury induced by middle cerebral artery occlusion was mitigated which was linked to increased survival rates, reduced infarct size, and reversed cognitive impairment [52]. Additionally, Peng et al. accessed publicly available genome-wide association studies of European ancestry (*n* > 198,000 adults) and reported a negative correlation between blood acetate and ischemic stroke using linkage disequilibrium score regression analysis [52].

In older stroke patients (*n* = 37; >65 years), Yuan et al. noted positive correlations for blood acetate and vascular endothelial growth factors, which are indicators of patient survival and neurological recovery [53]. In aged male C57BL/6 mice subjected to distal middle cerebral artery occlusion surgery, Yuan et al. noted that fecal microbiota transplantation from healthy young or aged mice three days prior to surgery had differing impacts on stroke recovery, which were directly related to acetate concentrations in the feces of the donors [53]. Fecal acetate was about 40% greater in the young donors versus older donors, which mirrored the differences in blood acetate levels in the stroke mice receiving either young or old fecal microbiota transplantation. Similarly, cortical penumbra microvessel density was about 30% greater in the stroke mice receiving young versus old fecal microbiota transplantations at 28 days post stroke, and improvements in functional measures post-stroke followed this same pattern [53]. An earlier investigation in transgenic mouse models provided evidence that microbiota-derived acetate was responsible for these protective effects by replenishing acetyl-CoA to fuel the TCA cycle [54]. These observations are correlational and not causal but support the need for future clinical investigations to clarify the potential health benefits of oral acetate supplementation in stroke recovery.

There are two small pilot RCTs in healthy adults demonstrating promising effects of daily vinegar ingestion on mood states and depression scores. During the COVID-19 lockdown, self-reported depression scores improved an average of 27% in college students randomized to the liquid vinegar arm (1.5 g acetic acid ingested daily for four weeks) compared to a slight worsening of depressive symptoms in the control arm ingesting commercial vinegar pills (0.015 g acetic acid ingested daily for four weeks; *p* ≤ 0.006) [55]. This was a small (*n* = 27), open-label investigation. However, the pills had a strong acidic/vinegar odor, and participants were unaware of the large difference in acetic acid content of the liquid versus pill treatments. In a follow-up study using a similar protocol (*n* = 28), improvements in depressive symptoms were again noted following liquid vinegar ingestion (2.95 g acetic acid daily for four weeks) compared to vinegar pill ingestion [56]. Metabolomic analyses of blood samples collected at baseline and at week four along with the generated KEGG pathway diagram revealed significant upregulation of the nicotinamide adenine dinucleotide (NAD) to nicotinamide pathway after adjusting for multiple comparisons using the False Discovery Rate method. Although not directly measured in this report, the NAD salvage pathway is linked to sirtuin-1 activation, which, as outlined below, promotes mitochondrial energetics.

### 5.2. Acetylation/Deacetylation Reactions

**Figure 4:**
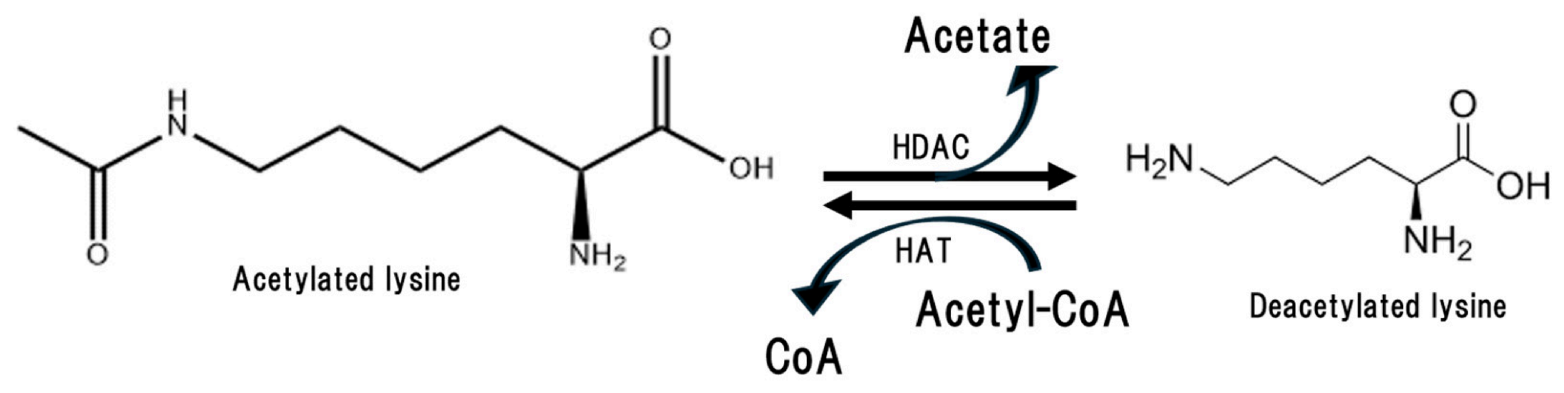
Lysine deacetylation as an example of acetylation/deacetylation reactions. HDAC: histone deacetylase; HAT: histone acetyltransferase.

Protein and amino acid acetylation is a reversible, post-translational modification where an acetyl group from acetyl-CoA is transferred to the *N*-terminus or lysine residues altering metabolite function to address a variety of metabolic needs (Figure 4) [57,58]. Acetylation/deacetylation reactions are about as widespread as phosphorylation/dephosphorylation reactions across all cells controlling metabolic activities and are balanced by the availability of acetyl-CoA, which functions as the acetyl donor, and the activity of deacetylases [59,60]. In tandem, the acetyl-CoA/acetate ratio acts as a nutrient sensing system for energy homeostasis and influences acetylation/deacetylation reactions to impact a wide range of enzyme activities in mouse models and in humans [61,62]. Elevated levels of acetyl-CoA indicate energy sufficiency and promote acetylation; whereas, in times of energy stress, hepatic production of acetate from fatty acids is stimulated and the acetate is sent to extrahepatic tissues, particularly the brain, to fuel energy production [62]. In this context, the authors suggested that acetate takes on a ‘ketone-like’ role to fuel the brain in times of stress [12,62].

The sirtuins, a family of seven NAD-dependent deacetylases, represent the primary deacetylases in mammalian systems [63]. Sirtuin 3 (SIRT3) is a prominent deacetylase in tissues with high mitochondrial content such as brain, and its activity promotes neuroprotective activities and mitochondrial energetics [64,65]. For example, in cell culture mitochondria SIRT3 deacetylates acyl-CoA short chain synthetase 2 to enhance the conversion of acetate into acetyl-CoA which can promote the TCA cycle and ATP production [65]. In mouse models, stressors linked to brain injury or neurodegenerative disorders create inflammatory milieus that block SIRT3 leading to destabilized mitochondria and reduced ATP production [66]. Recent clinical data revealed reductions in SIRT3 concentrations in patients with Parkinson’s disease (PD), and using mouse models of PD, the investigators were able to link reductions in SIRT3 to the mitochondrial dysfunction that is associated with dopaminergic neuron degeneration [67]. Similarly, SIRT1 activity was blocked in mice subjected to a 14-day-long chronic restraint stress model to induce a depression-like syndrome, and this deficiency was associated with reductions in mitochondrial energy production [68]. Acetate supplementation in rats was demonstrated to raise tissue levels of acetyl-CoA two-fold within 30 min [69], and evidence is mounting that dietary acetate attenuates neuroinflammation and promotes energy production through the TCA cycle [69,70,71].

**Figure 5:**
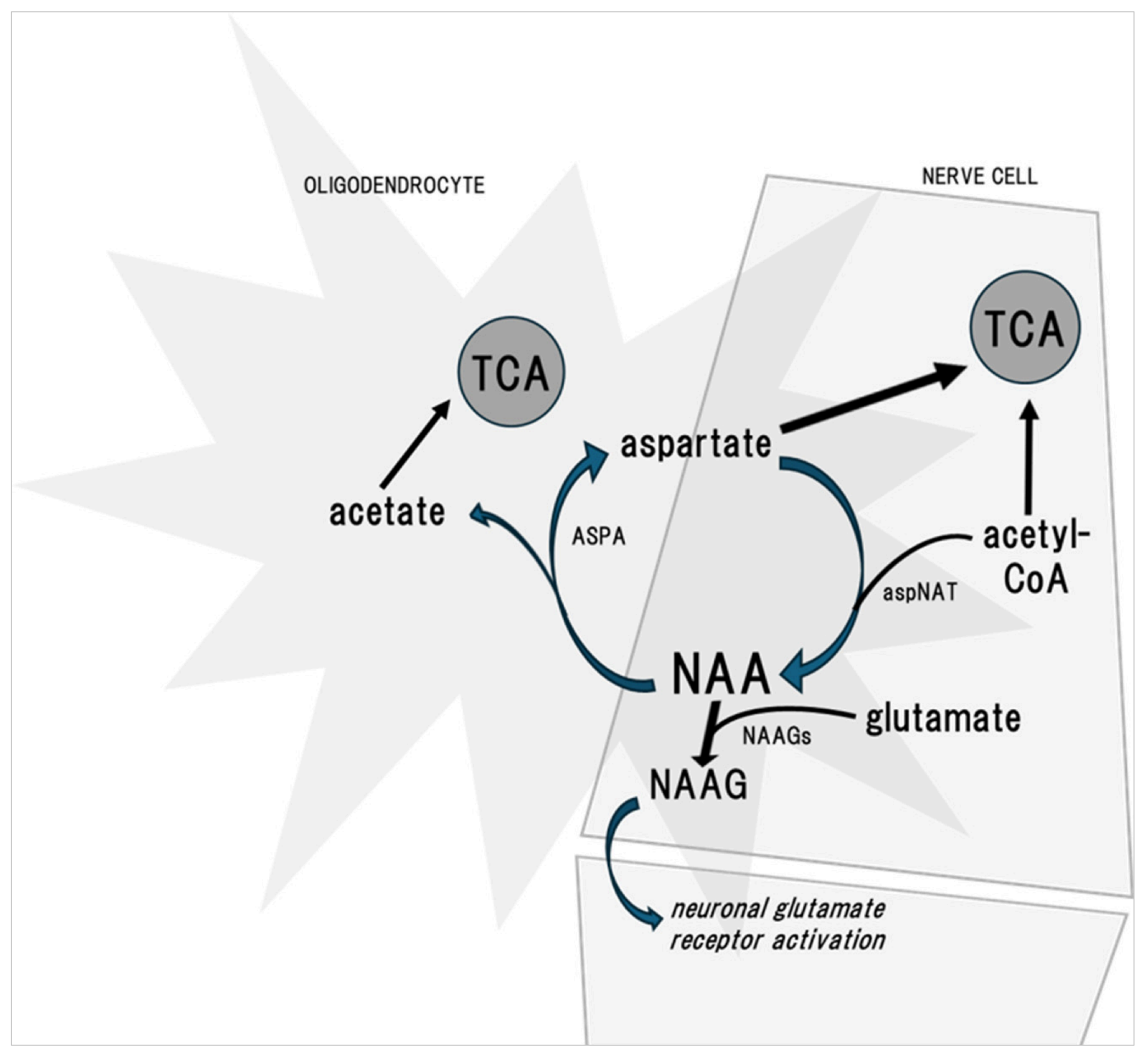
Conceptual summary of *N*-acetyl-aspartate (NAA) metabolism in the central nervous system. Acetyl coenzyme A (CoA) in neuronal mitochondria can enter the tricarboxylic acid (TCA) cycle for energy production or converted to NAA via aspartate *N*-acetyltransferase (aspNAT). NAA transported to oligodendrocytes is broken down to acetate via aspartoacylase (ASPA) and used for energy production or perhaps protein, fatty acid/myelin lipid synthesis. Neuronal NAA can also combine with glutamate to produce the neurotransmitter *N*-acetylaspartylglutamate (NAAG) via NAAG synthetase (NAAGs). NAAG plays a protective role against hyperactivity and neurodegeneration via interaction with glutamate receptors. (Adapted from [73]).

The acetylated amino acid, *N*-acetylaspartate (NAA) is the second most abundant amino acid in the central nervous system after glutamate [72]. It is produced in neuronal mitochondria by combining the amino acid aspartate and acetyl-CoA, catalyzed by the enzyme aspartate *N*-acetyltransferase (aspNAT). NAA can freely move from neurons to myelin-producing oligodendrocytes and following deacetylation by aspartoacylase (ASPA), NAA is a source of acetic acid to facilitate brain energetics and the production of myelin (Figure 5). As such, NAA is considered an energy reservoir in the brain and a key promoter of cognition and high-order brain function [72,73,74]. Varfolomeev et al. utilized functional magnetic resonance imaging of human brain and nuclear magnetic resonance technology to eloquently link brain stimulation, e.g., hemodynamic responses to external stimuli, with the deacetylation of NAA [75].

NAA can also combine with the amino acid glutamate to form the neuropeptide *N*-acetylaspartylglutamate (NAAG), a neurotransmitter and neuromodulator. NAAG plays a key role in the regulation of glutamate release, thereby benefiting neurodegenerative disorders linked to excitotoxicity from excessive glutamatergic signaling [76]. Reductions in NAA synthesis have been associated with neurodegenerative conditions in both mouse models and humans that include Alzheimer’s disease and Parkinson’s disease [41,77,78], a consequence of reduced levels of NAAG [79].

Canavan disease is a rare autosomal recessive disorder caused by mutations in the gene coding ASPA; hence, the deacetylation of NAA is impacted [80]. The major feature of Canavan disease is the lack of proper myelin formation during early development leading to rapid neurological decline. A pre-clinical investigation demonstrated that oral administration of acetate in the form of glyceryltriacetate (GTA) to tremor rat pups, a rat model of Canavan disease, significantly improved treadmill performance after four months as well as locomotion and exploration later in life [81]. The improvements in motor functions were positively correlated with decreased vacuolation in the cerebellum/brain stem and spinal cord [81]. In two human infants with Canavan’s disease (8 and 12 months of age), GTA treatment did not improve motor function; however, the GTA dosing was well tolerated, and in one child axial and limb muscle tone noticeably improved after 4.5 months of treatment [82]. The investigators recommended future investigations in patients below three months of age to evaluate the long-term efficacy of this therapeutic approach [82].

## 6. Vinegar as a Dietary Source of Acetic Acid

Vinegar has been a key ingredient of cuisines globally since antiquity. Likely discovered accidentally when wine was left in open air, the value of vinegar for food preservation and health were eventually recognized. Vinegar production is a two-step biological process in which sugars from fruits or grains are processed by yeast to produce alcohol and carbon dioxide. Acetic acid bacteria, of the family Acetobacteraceae, ferment alcohol to produce acetic acid [83]. Historical accounts suggest that Hippocrates, the father of modern medicine, widely utilized vinegar around 400 B.C. to prevent infection at surgical sites and help heal wounds, a reflection of acetic acid’s broad-spectrum antimicrobial and antiseptic properties. He also prescribed a tonic of vinegar and honey, known as oxymel, to treat coughs and respiratory conditions. Vinegar teas were prescribed to aid the diabetic condition as early as the 1700s [84]. In the 1800s a popular vinegar drink, Switchel, was believed to alleviate thirst and fatigue when laboring during hot weather [85]. These folk remedies may have scientific plausibility related to acetate metabolism; however, the clinical data discussed in this review are not definitive and should be viewed with caution.

Published reviews on this topic suggest that vinegar’s impacts on blood glucose and lipid regulation and weight loss are likely driven by acetic acid [86,87]. An oral dose of acetic acid in the range of 500–750 mg, equating to 2–3 teaspoons liquid vinegar, demonstrated antiglycemic effects in healthy adults and in patients with diabetes based on several small pilot RCTs [88,89]. Higher dosages (1500–3000 mg daily over time) were shown to reduce fasting glucose concentrations as well as reduce blood triglycerides and cholesterol, blood pressure, and adiposity [26,90,91,92]. A recent meta-analysis incorporating 25 clinical trials in adults concluded that vinegar ingestion may exert beneficial effects on cardiometabolic risk factors and reported the highest effect sizes for adults with type 2 diabetes in comparison to healthy adults or adults with obesity [36]. Effect sizes were also consistently larger for the studies of longer duration (>12 weeks). Hence, the efficacy of vinegar ingestion for improving health outcomes may depend on individual metabolic profiles and the duration of use.

Since vinegars, by definition, are watery liquids composed of 4% to 8% acetic acid, all commercial vinegars are a source of acetic acid. Vinegar acidity is stated on the label, and the acetic acid content of a given measure can be calculated. For example, vinegars at 5% acidity would contain 750 mg acetic acid per tablespoon (15 g × 0.05). When consumed as a condiment, the amount of vinegar per serving (*w*/*w*) may vary from 20% for dill pickles to 27% in commercial vinaigrette dressings and 40% in yellow mustard. Fermented products such as kombucha tea can be a source of acetic acid, although these levels can vary based on fermentation methods. One recent RCT in healthy adults did not observe any differences between groups for anthropometric, metabolic, or immune parameters following an 8-week intervention: 16 ounces of kombucha tea (75 mg acetic acid) daily versus control [93]. However, a second RCT in overweight adults recorded favorable shifts in the inflammatory profile and increased diversity in the oral microbial community in the tea group versus controls following a 12-week intervention (200 mL kombucha tea containing 680 mg acetic acid daily) [94].

It is important to recognize that vinegars and other fermented products contain a myriad of bioactive compounds such as polyphenols and organic acids, reflecting constituents of the base product, that are possibly partially responsible for healthful outcomes when these foods are ingested. Unlike the acetic acid content, which is stable across vinegars, the content of these bioactives vary by type of vinegar and the fermentation process, further complicating the interpretation of the clinical data. For example, in one report the content of the polyphenol gallic acid, a strong antioxidant, in vinegars varied from 0.8 to 6.0 ppm for apple and grape vinegars respectively produced using industrial methods (e.g., rapid fermentation, within hours, using submerged culture generators and high-speed aeration) [95]. Yet, for artisanal apple vinegars (e.g., slow natural surface or barrel fermentation, taking months or years) the gallic acid content was 61.2 ppm [95].

When consumed at doses up to two tablespoons, a systematic review of clinical trials in humans (thirteen trials reviewed with nearly 500 participants) concluded that vinegar ingestion appeared safe, although patients with insulin-dependent diabetes should be aware of the risk for greater frequency of hypoglycemia [96]. A recent meta-analysis encompassing 1320 participants across twenty-five trials noted that mild side effects (throat and gastrointestinal discomfort) were reported in three of the trials examined [36]. Vinegar should not be consumed undiluted due to risk for aspiration into lungs or irritation of the oral mucosa [97]. In patients with type 1 diabetes and diagnosed diabetic gastroparesis, vinegar ingestion (30 mL) prior to meal ingestion (300 g rice pudding) slowed gastric emptying 37% (*p* < 0.05; measured using ultrasonography) compared to water ingestion [98]. This worsening of gastroparesis may accentuate feelings of nausea, belly bloating and pain, fullness, and acid reflux in these patients. Gastroparesis in this population may be life-threatening if food is unable to pass into the small intestine. There is also evidence that the regular ingestion of vinegar as a diluted drink can adversely impact tooth enamel [99,100]. Consequently, the long-term safety of vinegar ingestion as a daily supplement remains unknown due to a lack of large, extended human clinical trials. Caution is due as the risk of side effects remains a possibility.

The taste of vinegar is unpleasant to many, which may have fueled the popularity of commercial vinegar pills, a market that is forecast to reach 2.27 billion by 2031 [101]. However, the acetic acid content for many of these preparations is less than 40 mg, a level well below the amounts used in clinical trials. Acetate pills are not currently marketed as a vinegar substitute, and few clinical trials have examined the efficacy of acetate supplementation in humans for the health benefits described herein. One early study compared the effect of sodium acetate (providing 750 mg acetate) versus liquid vinegar (750 mg acetic acid) on the 2 h postprandial glycemic response to a carbohydrate load in patients with type 2 diabetes [86]. The antiglycemic effect of sodium acetate was less than one-third of that noted for liquid vinegar. Whether acetate supplementation would amplify the systemic actions of acetate noted above for brain and muscle in humans is unknown.

## 7. Limitations and Caveats

This narrative review synthesized a diverse literature to provide a general overview of the metabolic effects of acetate, acetic acid, and vinegar ingestion on health parameters linked to chronic conditions including obesity, diabetes, and neurological disorders. Much of the evidence discussed was from preclinical trials that have yet to be replicated in humans. Moreover, the RCTs conducted to date in this topic area were small, underpowered studies of short duration, factors that greatly limit data interpretation due to reduced statistical power, increased sampling error, and the inability to reliably generalize the findings to a broader population. Vinegar and acetic acid/acetate dosages and formulations differed from study to study further confounding interpretation.

Finally, the author’s long-standing focus on vinegar research may be perceived as a potential conflict of perspective or source of bias. It is hoped that this interpretation of the literature sets the stage for future research to clarify the role of vinegar ingestion in health promotion and as a nutritional intervention to complement conventional medical treatments.

## 8. Conclusions

Acetate holds a prominent role in cellular metabolism by promoting tissue energetics and healthy brain function and stabilizing blood glucose concentrations. Acetate is produced endogenously in tissues and supplies 6–7% of energy expenditure in humans at rest. Additionally, acetic acid is the most abundant postbiotic produced by the gut microbiome and its conjugate base, acetate, is the primary short chain fatty acid in the systemic circulation. Acetate has a critical role in host homeostasis, serving as a metabolic substrate for skeletal muscle and a signaling molecule in the central nervous system. Vinegar is a dietary source of acetic acid, providing 750 mg per tablespoon at 5% acidity. Emerging clinical research in patient populations and healthy adults is suggestive of health benefits associated with vinegar ingestion; however, this literature is sparce, and the published reports were mainly conducted in the USA and Iran and displayed a high degree of heterogeneity in study quality, vinegar preparations and protocols, outcome measurements, and statistical variation. Well-designed investigations with large sample sizes and long duration are needed to verify these initial reports and identify the specific metabolic processes linking exogenous acetic acid with health outcomes. Future trials could address the standardization of acetate and vinegar dosage; differences among commercial vinegar preparations; the influence of gut microbiota composition on individual responses; personalized nutrition approaches; development of acetate-based therapeutic formulations; and identification of biomarkers to monitor treatment response.

## Data Availability

No new data were created or analyzed in this study. Data sharing is not applicable to this article.

## Abbreviations

The following abbreviations are used in this manuscript:

SCFA	Short chain fatty acids
RCT	Randomized controlled trial
TCA	Tricarboxylic acid cycle
ATP	Adenosine triphosphate
CoA	Coenzyme A
AMP	Adenosine monophosphate
AMPK	AMP-activated protein kinase
GLUT	Glucose transporter type
PPARα	Peroxisome proliferator-activated receptor α
ACO	Acyl-CoA oxidase
CPT1	Carnitine palmitoyl transferase 1
UCP2	Uncoupling protein 2
VFA	Visceral fat area
SFA	Subcutaneous fat area
PCr	Phosphocreatine
AD	Alzheimer’s disease
NAD	Nicotinamide Adenine Dinucleotide
SIRT	Sirtuin
PD	Parkinson’s disease
NAA	*N*-acetylaspartate
aspNAT	Aspartate *N*-acetyltransferase
ASPA	aspartoacylase
NAAG	*N*-acetylaspartylglutamate
GTA	glyceryltriacetate
HDAC	histone deacetylases
HAT	histone acetyltransferase
